# Prevalence and Risk Factors of Non-communicable Diseases in an Adult Tribal Population of Kanyakumari District, India: A Cross-Sectional Study

**DOI:** 10.7759/cureus.107773

**Published:** 2026-04-26

**Authors:** Vaisakh GT, Jayasree CS, Vishnu G Ashok, Bhuvanesh AK, Liji Anna Varghese

**Affiliations:** 1 Community Medicine, Sree Mookambika Institute of Medical Sciences, Kulasekharam, IND; 2 Community Medicine, Tirunelveli Medical College, Tirunelveli, IND

**Keywords:** hypertension, ncds, physical activity, prevalence, tribal population

## Abstract

Background

The broad category of illnesses known as non-communicable diseases (NCDs) includes conditions such as diabetes, cancer, heart disease, and chronic respiratory disorders. It causes million deaths worldwide each year, or of all fatalities. The concerning rise in NCDs is thought to be caused by the increasing prevalence of anthropometric and behavioural risk factors for these lifestyle diseases. Due to poor diet, greater exposure to harmful addictions such as tobacco, and lack of access to healthcare services, NCD rates are steadily rising among India’s marginalised people (tribal). In order to develop effective control strategies for these illnesses, it is crucial to monitor changing risk factors.

Objectives

The study aims to estimate the prevalence and risk factors of NCDs among adults in Pechiparai, a tribal area of Kanyakumari district, Tamil Nadu, India, and to determine the association between socio-demographic factors and risk factors of NCDs among the study participants.

Methodology

A community-based cross-sectional study was conducted from March 2021 to September 2022 among 420 adults (>18 years) residing in Pechiparai village, using simple random sampling. Data were collected through a pre-tested, interviewer-administered, semi-structured questionnaire after obtaining informed consent. Information on socio-demographic variables, NCD prevalence, and lifestyle risk factors was recorded. Blood pressure measurements were taken and classified according to the Joint National Committee on Prevention, Detection, Evaluation, and Treatment of High Blood Pressure (JNC 7) criteria. Microsoft Excel (Microsoft® Corp., Redmond, WA, USA) was used to enter the data, while IBM SPSS Statistics for Windows, Version 20 (Released 2011; IBM Corp., Armonk, NY, USA) was used for analysis. Chi-square tests and descriptive statistics were used, with a 95% confidence level.

Results

The mean age of participants was 44.92 ± 13.39 years, with females constituting 225 (53.6%). The total prevalence of hypertension was 216 (51.4%), including 102 (24.3%) newly detected cases. The prevalence of known diabetes mellitus was 70 (16.7%). Among the hypertensive participants, smoking was reported by 63 (51%) (majority beedi users), and 60 (57%) consumed alcohol. Extra salt intake was observed in 118 (75%). Among the hypertensive participants, hearing problems in 43 (61%) and visual impairment in 23 (55%) were other reported NCD-related conditions. A significant association was observed between hypertension and family history of NCDs (χ² = 21.52, p < 0.001). Extra salt intake showed a strong association with hypertension (χ² = 61.1, p < 0.001), with a notably higher prevalence among those reporting extra salt consumption (118, 75%) compared to those who did not (98, 37%).

Conclusion

The study revealed a high burden of hypertension and a significant prevalence of diabetes and behavioural risk factors among the tribal population of Pechiparai. The findings highlight the urgent need for targeted screening, health education, and strengthened implementation of NCD control programs in tribal areas to reduce morbidity and prevent future complications.

## Introduction

Non-communicable diseases (NCDs) consist of a vast group of diseases such as cardiovascular diseases, cancer, diabetes, and chronic respiratory diseases [1]. Globally, they kill 41 million people every year, equivalent to 71% of all deaths. Among these, about 15 million people belong to the age group 30 to 69 years, 85% of whom are premature deaths, mostly occurring in low- and middle-income countries (LMICs) [2]. The burden of NCDs such as diabetes and coronary heart disease (CHD) is increasing both globally and in India [3]. The rising prevalence of behavioural and anthropometric risk factors for these lifestyle diseases is postulated to be the cause of the alarming increase in NCDs [4]. In both low-resource and middle-resource nations, they are among the world’s worst public health issues in the 21st century, leading to poor social development, poor health, economic loss, death, and a lower quality of life [5,6].

The World Health Organization's (WHO) Global Status Report (GSR) on NCDs states that over 40% of the 38 million annual deaths from NCDs were preventable and premature [7]. The Asia Pacific region is experiencing a rapid increase in NCD-related deaths, with the highest worldwide increment in total mortality in a 10-year time frame (2005-2015) observed in the South-East Asia and Western Pacific regions, with a 21% increase in the South-East Asia region [8] and 12.3 million deaths in the Western Pacific region. NCD deaths are projected to increase by 15% between 2010 and 2020 [9].

The WHO projects that by 2030, NCDs will account for over 75% of all deaths in India [10]. It was specifically added to the resolutions adopted at the United Nations General Assembly's third high-level meeting (2018) on the prevention and control of NCDs [11]. NCD death rates are greater in LMICs than in high-income countries (HICs), even after reflecting changes in population age patterns [12].

In a vast nation like India, there are regional variations that are impacted by factors such as nutrition, culture, and socioeconomic position, even while the general tendency shows an increase in common adverse factors [13]. Lifestyle diseases can be a greater threat to many states, particularly Kerala and Tamil Nadu, which have been performing well in terms of health indicators [14].

Before the National Rural Health Mission (NRHM) was launched, the National Commission on Macroeconomics and Health (NCMH) under the Ministry of Health and Family Welfare (MOHFW), India, predicted a sharp rise in NCDs [15]. Previous research revealed that the sample population had a high burden of risk factors for NCDs, suggesting that the epidemiology of non-communicable illnesses in India is evolving [16]. Risk factor clustering was quite prevalent, and the majority of adults (83%) had at least one NCD risk factor [17].

NCD rates are rising steadily, even in India's rural areas, and they are now the country's leading cause of death. There is growing evidence that marginalised groups are more vulnerable to NCDs because they have less access to health care, are more likely to be exposed to harmful addictions like tobacco, and eat poorly [18]. High prevalence of hypertension and other NCD risk factors is found in India's tribal population [19].

The tribals of India, constituting 8.2% of the total population, belong to around 698 communities or clans. Of these, 75 groups are classified as “Primitive Tribal Groups.” The identified groups in Tamil Nadu are Kodas, Todas, Irulas, Kurumbas, Badagas, Kattunayakan, and Paliyan [20,21]. The concept of tribe had emerged in India before independence. Gradually, the reservation concept emerged, and then the idea of the Scheduled Tribe in independent India [22]. Because of their simple lifestyle, good eating habits, intense physical labour, and cleaner surroundings, the tribal population is often thought to have a low prevalence of NCDs [23].

One of the most vulnerable and marginalised segments of Indian society is the tribal community [24,25]. These areas are neglected because of extremely inadequate health care infrastructure, healthcare expenditures, hospital accessibility, and health manpower [26]. Since there are only limited studies based on tribal health, the present study is being planned to assess the prevalence of NCD risk factors among adults of the tribal population in Kanyakumari district.

## Materials and methods

This cross-sectional study was conducted among adults aged more than 18 years of both genders residing in Pechiparai village, located in the Thiruvattar block of Kanyakumari district, Tamil Nadu, India, from March 2021 to September 2022. The study protocol was approved by the Institutional Ethics Committee of Sree Mookambika Institute of Medical Sciences, Kulasekharam, India (Approval No: SMIMS/IHEC No: 1/Protocol no: 36/2021). Prior to data collection, informed consent was obtained from all participants after explaining the purpose of the study, and confidentiality and privacy were strictly maintained throughout the process.

The study included adult males and females aged above 18 years who had been residing in the study area for at least five years. Individuals who were severely ill (such as bedridden patients or those with congenital disorders) and those who did not provide consent were excluded from the study.

The sample size was calculated based on the study conducted by Sajeev and Soman [19] among the Kani tribe; the prevalence of hypertension was 48.3%. The sample size was calculated using the formula $n = \frac{Z_{\alpha}^{2} p q}{L^{2}}$, where Zα = 1.94, Zα² = 3.84, p = prevalence of HTN in the selected study was 48.3%. So, $p = 48.3\%, \; q = 100 - p = 100 - 48.3 = 51.7\%$.

With a relative precision of 10%, $L = 10\% \text{ of } P = 10\% \times 48.3 = 4.83, \quad n = \frac{(1.96)^2 \times 48.3 \times 51.7}{(4.83)^2} = 411$, approximated to 420.

The calculated sample size was 411, which was rounded off to 420. A simple random sampling technique was employed to select participants. Households were chosen using a random number generation method, and eligible adults from the selected households were interviewed. Houses without eligible adults were skipped, and the survey continued until the required sample size of 420 participants was achieved.

Data were collected using a pre-tested and validated semi-structured interview schedule, administered through face-to-face interviews. The self-prepared questionnaire was translated into the local languages (Malayalam and Tamil) and back-translated to ensure validity (see Appendix 1). It included details on socio-demographic characteristics, prevalence of NCDs, and associated risk factors.

The collected data were entered into Microsoft Excel 2013 (Microsoft® Corp., Redmond, WA, USA) and analysed using IBM SPSS Statistics for Windows, Version 20 (Released 2011; IBM Corp., Armonk, NY, USA). Descriptive statistics were expressed as frequencies, percentages, and mean ± standard deviation. The association between NCDs and socio-demographic variables was assessed using the Chi-square test, and a p-value of less than 0.05 was considered statistically significant.

## Results

A cross-sectional study was conducted at Pechiparai village panchayat under Thiruvattar Block. A total of 420 adult tribal participants were included in the study using a simple random sampling technique. Table 1 shows that the mean age of the study participants was 44.92 ± 13.39 years. Most of the study population belonged to the age group of 41-50 years (130, 31%), followed by 31-40 years (105, 25%), with the least in the age group of 20 years or less (6, 1.4%). Female participants were more (225, 53.6%) compared to males (195, 46.4%). Of the total participants, most were currently married (369, 87.9%), and 12 (2.9%) were widowed. A total of 148 (35.2%) of the study participants belonged to the middle class, and 146 (34.8%) to the lower middle class, followed by 88 (21%) in the lower class. 

**Table 1:** Demographic Profile of the Participants (n = 420)

Variables		Frequency	Percentage
Age	≤ 20	6	1.4
	21-30	59	14.1
	31-40	105	25
	41-50	130	31
	51-60	72	17.1
	61-70	30	7.1
	> 70	18	4.3
Gender	Male	195	46.4
	Female	225	53.6
Religion	Hindu	409	97.4
	Christian	7	1.7
	Muslim	4	1
Marital status	Married	369	87.9
	Unmarried	39	9.3
	Widowed	12	2.9
Type of family	Nuclear	398	97.8
	Joint	18	-
	Three generational	4	1
Place of work	Self employed	223	74.1
	Government sector	36	12
	Private sector	42	13.9
Socio-economic status	Upper	5	1
	Upper middle	35	8.1
	Middle	148	35.2
	Lower middle	146	34.8
	Lower	88	21

Prevalence of NCDs

**Figure 1:**
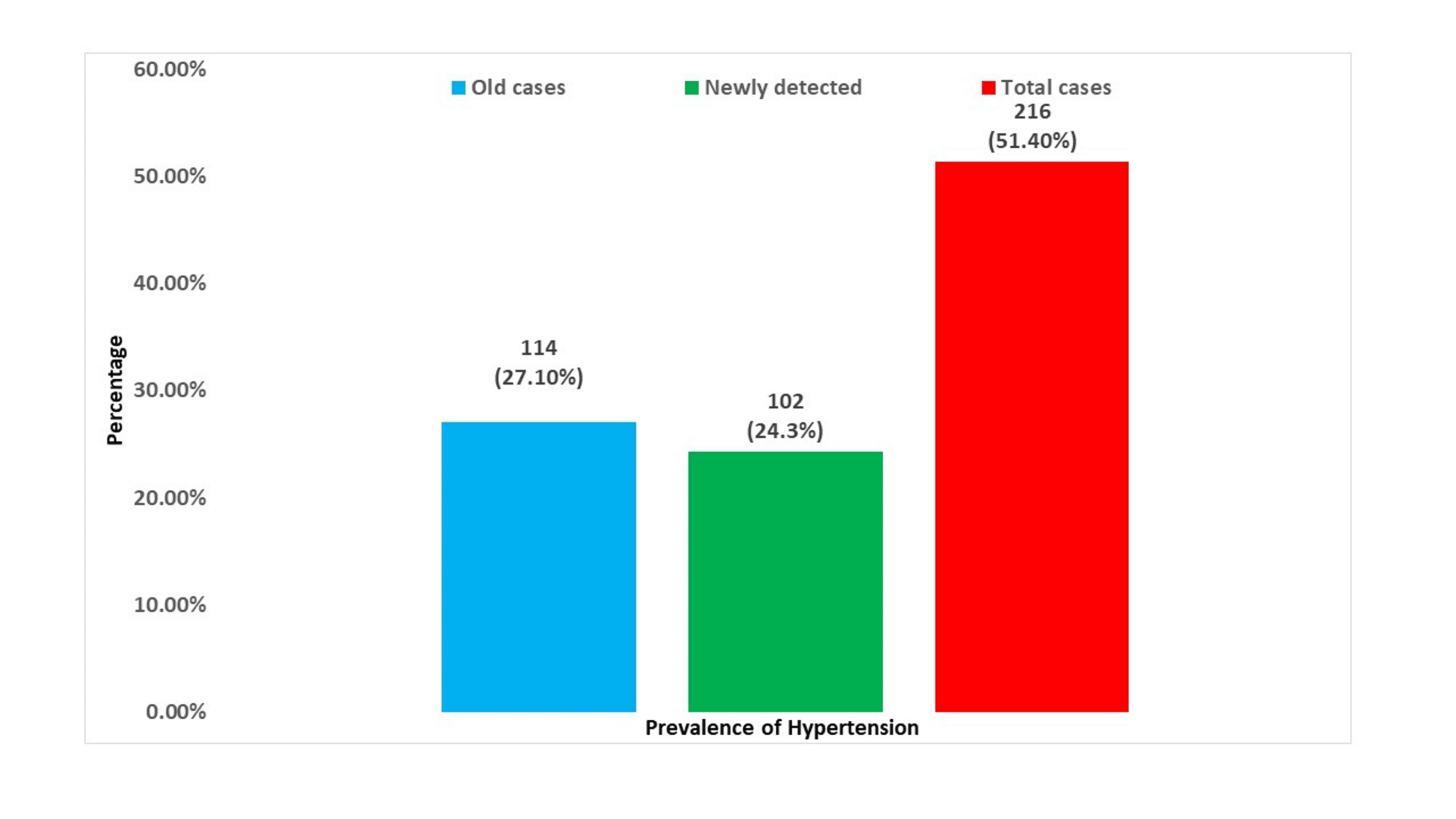
Prevalence of Hypertension (n = 420)

The prevalence of self-reported hypertension was 114 (27.10%). According to JNC 7 classification, 102 (24.3%) were newly detected to have hypertension. Therefore, the total prevalence of hypertension among our study population was 216 (51.40%) (Figure 1).

The prevalence of known cases of diabetes mellitus was 70 (16.7%). The prevalence of other NCDs, such as hearing problems, among the study participants was 71 (16.9%). A total of 42 (10%) had visual impairment. Of the total percentage of vision problems, the majority of participants had refractive errors (120, 28.6%). Two participants were diagnosed with prostate cancer, giving a prevalence of 2 (0.5%).

Table 2 shows the association between hypertension and selected demographic and socio-economic variables among the study participants (N = 420). Age was found to be significantly associated with hypertension (χ² = 39.03, p < 0.001), with a markedly higher prevalence among individuals aged ≥40 years (176, 62%) compared to those <40 years (40, 29%). Gender did not show a significant association with hypertension, as the prevalence was comparable among males (102, 52%) and females (114, 51%) (p = 0.737).

**Table 2:** Association of Hypertension With Demographic and Socio-Economic Variables *Statistically significant (p < 0.05).

Variables		Hypertension		χ²-value	p-value
		Yes, n (%)	No, n (%)		
Age	<40 years	40 (29%)	96 (71%)	39.03	<0.001*
	≥40 years	176 (62%)	108 (38%)		
Gender	Male	102 (52%)	93 (48%)	0.113	0.737
	Female	114 (51%)	111 (49%)		
Religion	Christian	2 (29%)	5 (71%)	1.49	0.474
	Hindu	212 (52%)	197 (48%)		
	Muslim	2 (50%)	2 (50%)		
Marital status	Married	201 (54%)	168 (46%)	13.92	0.001*
	Unmarried	9 (23%)	30 (77%)		
	Widowed	6 (50%)	6 (50%)		
Education	Illiterate	29 (76%)	9 (24%)	30.33	<0.001*
	Primary-middle school	100 (59%)	69 (41%)		
	High school & above	87 (39%)	126 (61%)		
Occupation	Farmer/Unskilled	116 (61%)	72 (39%)	25.67	<0.001*
	Skilled/Professional	20 (33%)	41 (67%)		
	Unemployed	60 (50%)	59 (50%)		
Place of work	Government	17 (47%)	19 (53%)	8.5	0.014*
	Private	13 (31%)	29 (69%)		
	Self-employed	186 (54%)	156 (46%)		
Socio-economic status	Upper & upper-middle	22 (58%)	16 (42%)	5.55	0.235
	Middle & below	194 (50%)	188 (50%)		

Religion was also not significantly associated with hypertension (p = 0.474). However, marital status showed a significant association (χ² = 13.92, p = 0.001), with a higher prevalence of hypertension among married individuals (201, 54%) compared to unmarried participants (9, 23%). Education level was significantly associated with hypertension (χ² = 30.33, p < 0.001), with the highest prevalence observed among illiterate participants (29, 76%) and a declining trend with increasing educational attainment.

Occupation demonstrated a significant association with hypertension (χ² = 25.67, p < 0.001). Participants engaged in farming or unskilled work had a higher prevalence, 116 (61%), compared to skilled or professional workers, 20 (33%). Place of work was also significantly associated with hypertension (χ² = 8.5, p = 0.014), with self-employed individuals showing a higher prevalence, 186 (54%), than those working in government or private sectors. Socio-economic status did not show a statistically significant association with hypertension (p = 0.235).

Table 3 presents the association between hypertension and lifestyle-related and other risk factors. A significant association was observed between hypertension and family history of NCDs (χ² = 21.52, p < 0.001), with a higher prevalence among participants without a family history (190, 56%) compared to those with a positive history (26, 32%).

**Table 3:** Association of Hypertension With Lifestyle and Other Risk Factors *Statistically significant (p < 0.05).

Variables		Hypertension		χ²-value	p-value
		Yes, n (%)	No, n (%)		
Family history of NCDs	Yes	26 (32%)	56 (68%)	21.52	<0.001*
	No	190 (56%)	148 (44%)		
Vigorous physical activity	No	149 (53%)	130 (47%)	11.03	0.004*
	Yes	67 (48%)	74 (53%)		
Fruit consumption	<3 times/week	21 (55%)	17 (45%)	10.04	0.040*
	≥3 times/week	195 (52%)	183 (48%)		
Vegetable consumption	<3 times/week	4 (27%)	11 (73%)	4.47	0.107
	≥3 times/week	212 (52%)	193 (48%)		
Junk food consumption	Frequent	8 (57%)	6 (43%)	15.9	0.001*
	Never/Seldom	208 (49%)	198 (51%)		
Carbonated drinks	Frequent	4 (40%)	6 (60%)	11.74	0.019*
	Never/Seldom	212 (52%)	198 (48%)		
Extra salt intake	Yes	118 (75%)	39 (25%)	61.1	<0.001*
	No	98 (37%)	165 (63%)		
Alcohol consumption	Yes	60 (57%)	46 (43%)	4.88	0.087
	No	156 (50%)	158 (50%)		
Smoking status	Current smoker	63 (51%)	60 (49%)	2	0.367
	No	153 (52%)	144 (48%)		
Passive smoking	Present	74 (61%)	47 (39%)	9.77	0.021*
Hearing impairment	Yes	43 (61%)	28 (39%)	6.41	0.040*
	No	173 (50%)	176 (50%)		
Vision problems	Yes	23 (55%)	19 (45%)	4.42	0.109
	No	193 (51%)	185 (49%)		

Vigorous physical activity showed a statistically significant association (χ² = 11.03, p = 0.004), with a higher prevalence of hypertension among individuals who did not engage in vigorous activity (149, 53%) compared to those who did (67, 48%). Fruit consumption was significantly associated with hypertension (χ² = 10.04, p = 0.040), whereas vegetable consumption did not show a significant association (p = 0.107).

Frequent consumption of junk food (χ² = 15.9, p = 0.001) and carbonated drinks (χ² = 11.74, p = 0.019) was significantly associated with hypertension. Extra salt intake showed a strong association with hypertension (χ² = 61.1, p < 0.001), with a notably higher prevalence among those reporting extra salt consumption (118, 75%) compared to those who did not (98, 37%).

Alcohol consumption and smoking status were not significantly associated with hypertension. However, passive smoking was significantly associated (χ² = 9.77, p = 0.021) with a higher prevalence among those exposed to passive smoking (74, 61%). Hearing impairment was also significantly associated with hypertension (χ² = 6.41, p = 0.040), whereas vision problems did not show a statistically significant association (p = 0.109).

## Discussion

The present study, conducted in a tribal area of Tamil Nadu, included participants with a minimum age of 19 years and a maximum of 88 years, resulting in a mean age of 44.92 ± 13.39 years. Similar studies among different ethnic groups in other states of India also support the relevance of our results. The mean age of the sample population of tribes in Assam was 38 ± 9.6 years in 2011 [16]. Sarma et al. reported a mean age of 42.5 years, with a standard deviation (SD) of 14.8 [17]. The statistics from a few studies indicate an age distribution of 25-34 years, whereas in our study it was 41-50 years [16,19]. In the present study, about 46.4% (195) were males, and 53.6% (225) were females. Sathiyanarayanan et al. reported that, among the tribal population in India, out of 952 interviewed individuals, 40.2% were males and 59.8% were females [27].

The majority of study participants (97.4%) were Hindus. According to a study conducted in Kerala by Negi et al., the vast majority were Hindu (W: 65.1% and M: 65.6%), followed by Muslims and Christians [28].

In the present study, most of the participants were currently married (87.9%), 2.9% were widowed, and there were no divorcees. A similar study by Ganie et al. found that, out of the study population, 71.8% were married, 25.2% were unmarried, 2.1% were widowed, 0.7% lived separately, and 0.2% were divorced [29].

In our study, the nuclear family was the most common type of family practised by the participants. A study on the Kashmiri tribal population by Ganie et al. found that the nuclear type of family (53.7%) was the most common [29]. Our study also showed that, among the study participants, the majority had education up to high school (33.3%), followed by middle school (26%), and only 9% were illiterate. Studies by Sathiyanarayanan et al. and Ganie et al., respectively, found that the illiterate population was 82.2% and 62.9% [27,29]. Our study focused on a population with a higher literacy rate, as reported in the 2011 census for Kanyakumari district [21].

In our study, the majority of the study participants were farmers (29.1%), and 28.3% were unemployed. Another study by Gupta et al. found that 13.6% were unemployed [30].

A high prevalence of hypertension was found in the present study (51.4%), of which 27.10% was self-reported and 24.3% was newly detected according to JNC 7 classification. The prevalence of known cases of diabetes mellitus was 16.7% among the study participants. Two participants were already diagnosed with prostate cancer, making the prevalence of cancer 0.5%. The prevalence of hearing problems among the study participants was 16.9%. About 10% had visual impairment.

According to a cross-sectional study conducted in Delhi, 18% of the participants had elevated fasting blood sugar levels. Over one-third of the subjects had aberrant lipid profiles and elevated systolic and diastolic blood pressure [10]. According to a study by Misra et al., 2.4% of hypertensives were under control, 17% were treated, and 24% were aware of their condition [16]. In a study by Sarma et al., the overall prevalence of elevated FBG was 19.2% (95% CI, 18.1 to 20.3), and elevated BP was 30.4% (95% CI, 29.1 to 31.7) [17].

A total of 3.8% of people had diabetes, with 77.8% of those cases being newly diagnosed. A study by Sathiyanarayanan et al. found that the overall prevalence of hypertension was 16.7%, of which 62.9% were newly detected [27].

Numerous studies have identified similar associations. For example, a study by Sathiyanarayanan et al. indicated that the presence of hypertension was correlated with age, gender, body mass index, and literacy status. Just eight individuals had both hypertension and diabetes. It was discovered that there was a statistically significant correlation (p = 0.05) between increasing age and diabetes. There were no other significant factors [27].

Another study conducted in a tribal population in Kerala showed that hypertension was significantly associated (p < 0.05) with higher age, male sex, low education levels, and tobacco intake [19]. In the present study, hypertension was found to be significantly associated with age, marital status, food habits, extra salt intake, and passive smoking. 

Limitation

The study had several limitations. Language barriers and reliance on self-reported data on diet and physical activity may have affected the accuracy of the findings. Being a cross-sectional study, it could not establish temporal relationships between socio-demographic factors and NCD risk factors, and clinical measurements like fasting blood sugar and repeated blood pressure recordings were not feasible. Additionally, the study was limited to one block with resource constraints and poor availability of medical records, which restricts the generalisability of the results.

Recommendations

The study emphasises the importance of promoting a healthy lifestyle among tribal populations through increased awareness, discouraging unauthorised medical practices, and encouraging a balanced diet and physical activity. It also highlights the need for multi-professional education in NCD care to improve understanding of disease progression and complications. Strengthening healthcare services by increasing NCD clinics, enforcing stricter regulations on tobacco and alcohol use, and enhancing IEC activities on healthy living are essential for effective prevention and control of NCDs.

## Conclusions

In conclusion, the study underscores that tribal populations are no longer protected from NCDs and, in fact, represent a vulnerable and underserved group with a dual burden of traditional and modern health risks. The high prevalence of undiagnosed hypertension and clustering of behavioural risk factors, such as tobacco and alcohol use, calls for urgent, targeted public health interventions. Bridging the gap in healthcare access and addressing social determinants such as education and socioeconomic status will be key to controlling the growing NCD burden in tribal populations. This study provides important baseline data for policymakers to design inclusive and region-specific intervention strategies.

## Appendices

Appendix 1: Questionnaire for participants

Non-communicable disease risk among adults

1. Name of the Block/Village Panchayat/Town Panchayat:

2. Age in completed years:

3. Religion: a) Hindu b) Muslim c) Christianity

4. Marital status: a) UnMarried b) Married c) Separated d) Divorced e) Widowed

5. Type of family: a) Nuclear b) Joint c) Extended family

6. Participants' education:

a) Illiterate b) Primary c) Middle SC d) High SC e) inter/diploma f) graduate g) professional

7. Participants' Occupation:

a) Professional b) Semi -Professional c) Clerical, Shop owner, Farmer

d) Skilled worker e) Semi-skilled worker f) Unskilled worker g)Unemployed.

8. Total family income/ month:_________ Total Family Members: _________

9. Place of Work: private or govt sector

10. Hypertension: yes/no, if yes, AYUSH/Allopathy

11. Diabetes: yes/no

12. History of any other non-communicable disease: yes/no, if yes AYUSH/Allopathy

13. Family history of non-communicable diseases: yes/no, if yes, AYUSH/Allopathy

Father: DM/SHT/Dyslipidemia/others

Mother: DM/SHT/Dyslipidemia/others

Sibings: DM/SHT/Dyslipidemia/others

14. Menopause: yes/no

15. Work involves vigorous activity. a)Yes b) No

16. Duration of vigorous-intensive activities at work on a typical day?

17. How much time do you spend walking

18. Time duration: bicycling per day?

19. Food frequency (Table 4)

20. Use of extra salt: yes/no

21. Have you ever consumed alcohol: a) yes, b) no. Age of starting: ___; Frequency: daily/2-3 times per week/weekly once/ occasionally/never

22. Why did you start consuming alcohol? Pick one

a) Peer pressure b) Curiosity c) Because you 'felt like it' (Bored) d) work stress

23. Do you feel you are a normal drinker? a) Yes b) No

24. Units of alcohol consumed? 1 or 2 / 3 or 4 / 5 or 6 / 7 to 9 / 10 or more

If yes, AYUSH/Allopathy

25. How often during the past years (Table 5)

26. How often during the past year have you been unable to remember what happened the night before because you had been drinking?

Never/Occasionally/Monthly/Weekly/Daily

27. Have you or somebody else been injured as a result of your drinking?

No, this has never happened/Yes, but not in the past year/Yes, during the past year

28. Has a relative, friend, doctor or health worker been concerned about your drinking or suggested you cut down?

No, never/Yes, but not in the past year/Yes, during the past year

29. Do you currently smoke? a) yes b) no

If no, have you smoked in the past? a) yes b) no. years since stopped:

30. What kind of smoking you do? a) cigarette b) pipes c) cigar d) hukkha e) snuff f) chewing g) dipping

31. At what age you started using it:

32. Who initiated you to use: a) family b) friends c) media d) others

33. How often do you smoke a) daily b) weekly c) occasionally

34. How much you smoke per day:

35. How soon after you wake up from bed do you usually have your 1st smoke:

36. Do you have any Intention to quit tobacco use: yes/no

37. Have you ever tried for quitting? If yes, how long and the reason.

38. Do any of your family members use tobacco? a) yes b) no c) don’t know

39. How often does anyone smoke inside your home? a) daily b) weekly c) monthly

40. Based on what you know or believe, does breathing others' smoke cause serious illness in non-users? a) yes b) no c) don’t know

41. Do you think tobacco causes cancer? a) Yes b) No

42. Which of the following are risk factors for developing cancers? a) over weight/obesity b) processed meats c) alcohol d) radiation e) all of the above

43. Do you have difficulty while talking on the phone or hearing the TV or radio? Yes/No

44. For the last 6 months, have you ever consulted an eye specialist: a) yes b) no. If yes, mention your problem:

45. Were you diagnosed with cancer? Yes/no. If yes, type: BP:____ GRBS: ____

**Table 4:** Food Frequency

Food items	Never	Seldom	1-2 times/mon	3-4 times/mon	2-3 times/wk	>3 times/wk
Fruits						
Vegetables						
Carbonated drinks						
Junk foods						

**Table 5:** How Often During the Past Years

	Never	Less than a month	Monthly	Weekly	Daily or almost daily
Not able to avoid drinking					
You failed to do what was normally expected					
Had a drink in the morning					
Had a feeling of guilt or remorse after drinking					
